# Diagnostic Accuracy and Colonoscopy-Sparing Potential of RAID-CRC Screen After a Positive Faecal Immunochemical Test

**DOI:** 10.3390/diagnostics16213655

**Published:** 2026-11-05

**Authors:** Cristina Regueiro-Expósito, Anais Crespo, Astrid Díez-Martín, Pedro Davila-Piñón, Joaquín Cubiella

**Affiliations:** 1 Grupo de Investigación en Oncología Digestiva-Ourense (GIODO), Fundación Biomédica Galicia Sur, 32003 Ourense, Spain; cristinaregueiroexposito@gmail.com (C.R.-E.); anais.crespo@iisgaliciasur.es (A.C.); astrid.diez@iisgaliciasur.es (A.D.-M.); pedro.davila@iisgaliciasur.es (P.D.-P.); 2 Research Section, Área Sanitaria de Ourense, Verín e O Barco de Valdeorras, Servizo Galego de Saude, 32003 Ourense, Spain

**Keywords:** colorectal cancer, screening, faecal immunochemical test, gut microbiota, diagnostic accuracy, triage, colonoscopy, RAID-CRC Screen

## Abstract

**Background/Objectives:** Faecal immunochemical test (FIT)-based colorectal cancer (CRC) screening generates many colonoscopy referrals without advanced neoplasia. We evaluated RAID-CRC Screen, a qPCR-based faecal bacterial signature, as second-line triage after positive FIT in the Galician population-based screening programme. **Methods:** This cross-sectional diagnostic accuracy study included participants with FIT ≥ 100 ng haemoglobin/mL and colonoscopy as reference standard. RAID-CRC Screen was analysed from residual FIT-buffer material. The primary analysis included evaluable assays and complete colonoscopy. An additional intention-to-diagnose analysis assigned non-evaluable results to colonoscopy referral. Comparisons with data-derived alternative FIT thresholds were exploratory. **Results:** Among 1291 participants in the primary analysis, 34 (2.6%) had CRC and 441 (34.2%) advanced neoplasia. RAID-CRC Screen was positive in 1087 (84.2%). Sensitivity was 94.1% (95% CI, 80.3–99.3%) for CRC and 87.5% (84.1–90.5%) for advanced neoplasia; specificities were 16.1% (95% CI, 14.1–18.2%) and 17.5% (95% CI, 15.0–20.3%), respectively. RAID-CRC Screen after FIT100 would avoid 204 colonoscopies (15.8%; 95% CI, 13.9–17.9%) while missing two CRCs and 55 advanced neoplasms. FIT118 alone would avoid 149 colonoscopies (11.5%), with CRC sensitivity of 100.0% (95% CI, 89.7–100.0%) and advanced neoplasia sensitivity of 92.3% (95% CI, 89.4–94.6%). In the intention-to-diagnose analysis (1312), including 21 non-evaluable results, colonoscopy reduction was 15.5% (95% CI, 13.6–17.6%), with the same two CRCs not referred. **Conclusions:** RAID-CRC Screen triage reduced modelled colonoscopy referrals at the cost of missed CRCs and advanced neoplasia. FIT118 alone avoided fewer colonoscopies while retaining all observed CRCs. These exploratory comparisons do not establish superiority or the safety of withholding colonoscopy.

## 1. Introduction

Colorectal cancer (CRC) remains one of the most frequently diagnosed malignancies and a leading cause of cancer-related death worldwide [1]. Population-based screening reduces CRC mortality and can also lower incidence through the detection and removal of advanced precursor lesions [2,3]. In Galicia, implementation of the organised faecal immunochemical test (FIT)-based screening programme was associated with a marked reduction in CRC mortality among the target population, supporting the effectiveness of this strategy in routine practice [4].

Most European organised programmes use a two-step pathway in which asymptomatic individuals complete a quantitative FIT, and those exceeding a predefined faecal haemoglobin threshold are referred for colonoscopy. FIT is non-invasive, acceptable, and scalable, but its sensitivity is greater for CRC than for advanced precursor lesions, and its positive predictive value is limited [5,6]. Consequently, a substantial proportion of FIT-positive participants undergo colonoscopy without evidence of CRC or advanced neoplasia. This creates pressure on endoscopy capacity and exposes participants to the burden and uncommon complications of an invasive procedure [7].

Because FIT provides a quantitative result, programmes may adapt the positivity threshold to match available colonoscopy capacity. Raising the threshold reduces referrals but inevitably decreases sensitivity, and the optimal balance varies according to population risk, sex, age, screening round, and programme characteristics [8,9,10]. During periods of constrained endoscopy capacity, threshold adjustment has therefore been used as an operational strategy, although it is a relatively non-selective means of prioritisation [11].

Gut microbial signatures have been proposed as complementary biomarkers that may refine risk among FIT-positive individuals. RAID-CRC Screen (GoodGut, S.L.U., Girona, Spain) is a qualitative test based on quantitative polymerase chain reaction (qPCR) measurements of selected faecal bacterial markers. An initial microbial signature was developed in symptomatic patients, and a separate screening-specific version was subsequently derived and validated in FIT-positive screening cohorts [12,13,14,15]. In these cohorts, RAID-CRC Screen reduced FIT false-positive results while maintaining high sensitivity for CRC and advanced neoplasia [12,13,14,15]. However, diagnostic performance may vary when a biomarker is transported to a new population and laboratory workflow. Independent, setting-specific validation is therefore required before incorporation into an organised screening programme.

The primary aim of this study was to externally assess the diagnostic accuracy of RAID-CRC Screen for CRC and advanced neoplasia among FIT-positive participants in the Galician population-based CRC screening programme. Secondary aims were to quantify the reduction in false-positive FIT referrals and colonoscopy demand, compare microbiota-based triage with alternative FIT thresholds, and explore performance according to sex and age.

## 2. Materials and Methods

### 2.1. Study Design and Reporting

We conducted a cross-sectional, post-market diagnostic accuracy study nested within the organised population-based CRC screening programme of Galicia, Spain (study code GG-RAIDCRC-1008), which was approved by the Local Institutional Review Board (code: 2023/243 on 27 June 2023). The study was performed at the Complexo Hospitalario Universitario de Ourense. Participants were recruited among subjects who underwent colonoscopy between December 2022 and October 2024, and the study was formally closed on 13 July 2026 after completion of sample processing and database quality control. The manuscript was prepared in accordance with the Standards for Reporting Diagnostic Accuracy Studies (STARD 2015) [16].

### 2.2. Screening Programme and Participants

The Galician programme invites asymptomatic individuals aged 50–69 years to complete FIT biennially. A faecal haemoglobin concentration of at least 100 ng Hb/mL buffer (equivalent to 20 micrograms Hb/g faeces for the OC-Sensor system, Eiken Chemical Co., Ltd., Tokyo, Japan) is considered positive, and participants with a positive result are referred for screening colonoscopy according to routine programme procedures.

Consecutive participants with a positive FIT who had undergone or were scheduled to undergo colonoscopy were assessed for eligibility. Exclusion criteria were withdrawal or death before completion of the study procedures; previous CRC, advanced adenoma, or inflammatory bowel disease; antibiotic treatment during the preceding month; severe comorbidity; gastrointestinal disease or surgery considered likely to alter intestinal integrity; age outside the programme range; and chemotherapy or radiotherapy during the preceding six months. All included participants provided written informed consent for use of residual FIT-buffer material and clinical data between 27 June 2023 and 13 December 2024.

### 2.3. Index Test: RAID-CRC Screen

Residual material from the FIT collection tube was stored at −20 degrees C immediately after faecal haemoglobin quantification until processing for RAID-CRC Screen analysis. The median storage duration was 273 days (IQR, 191–556). DNA was extracted using the DNeasy PowerSoil Pro kit (Qiagen, Hilden, Germany). The bacterial targets included in the commercial signature were quantified by qPCR using the RAID-CRC Screen qPCR kit. qPCR analyses were performed at the Complexo Hospitalario Universitario de Ourense between 4 October 2023 and 2 June 2025. Raw qPCR results were entered into the GoodGut-Test web platform, which applies the prespecified RAID-CRC Screen algorithm and generates a qualitative positive or negative result. The index-test threshold was not modified using the Galician dataset. Laboratory personnel were unaware of the colonoscopy and histopathology findings.

### 2.4. Reference Standard and Diagnostic Categories

Colonoscopy with histopathological assessment of resected or biopsied lesions was the reference standard. Endoscopists performed examinations in accordance with the quality procedures of the regional screening programme. Investigators reviewing colonoscopy and pathology records were blinded to RAID-CRC Screen results.

CRC was defined as invasive colorectal adenocarcinoma. Advanced adenoma was defined as a conventional adenoma measuring at least 10 mm, with a villous component or with high-grade dysplasia, including intramucosal carcinoma. Advanced neoplasia comprised CRC or advanced adenoma. Any colorectal neoplasia comprised CRC or any adenoma. Diagnostic categories were defined at participant level as non-mutually exclusive indicators of lesion presence; participants with CRC could also have synchronous adenomas or advanced adenomas.

### 2.5. Outcomes

The primary outcomes were the sensitivity and specificity of RAID-CRC Screen for CRC and advanced neoplasia within the FIT-positive cohort. Secondary outcomes included positive predictive value (PPV), negative predictive value (NPV), likelihood ratios, reduction in false-positive FIT results, the proportion and number of colonoscopies avoided, and corresponding performance for advanced adenoma and any colorectal neoplasia.

To assess the operational trade-off between colonoscopy demand and lesion detection, we evaluated FIT thresholds of 100, 118, 141, 155, 171, and 266 ng Hb/mL. For each threshold, two strategies were modelled: referral based on FIT alone and referral only when both FIT and RAID-CRC Screen were positive. The reference strategy was FIT100 followed by colonoscopy for all FIT-positive participants.

### 2.6. Sample Size

Based on the protocol’s performance targets of CRC sensitivity above 95% and a 15% reduction in colonoscopy referrals, and assuming a CRC prevalence of 2%, an absolute precision of 3 percentage points and a 90% confidence level, the protocol specified a minimum sample size of 1280 FIT-positive participants with a valid colonoscopy diagnosis.

### 2.7. Statistical Analysis

Continuous variables are presented as medians and interquartile ranges (IQRs), and categorical variables as counts and percentages. In accordance with the protocol, the primary analysis included participants with evaluable RAID-CRC Screen results and a valid colonoscopy-based reference diagnosis; incomplete colonoscopies were excluded. Two-by-two tables were constructed for each target condition to estimate sensitivity, specificity, positive predictive value and negative predictive value, with exact binomial 95% confidence intervals (CIs). Positive and negative likelihood ratios and their CIs were calculated using the logarithmic method. All performance estimates are conditional on a positive programme FIT result (≥100 ng Hb/mL).

The frequency of non-evaluable assay results was reported separately. An additional, non-prespecified intention-to-diagnose analysis included all participants with complete colonoscopy. Positive and non-evaluable RAID-CRC Screen results were assigned to colonoscopy referral, and negative results to no referral. Estimates from this analysis therefore describe the referral strategy rather than the assay alone.

Exploratory analyses evaluated alternative FIT thresholds, selected from the study dataset according to observed changes in CRC sensitivity, with and without RAID-CRC Screen triage. FIT-only strategies required FIT ≥ the specified threshold for referral; combined strategies additionally required a positive RAID-CRC Screen result. These analyses used the same population with evaluable assay results. Colonoscopies potentially avoided were expressed as a percentage of all participants in the corresponding analysis population. CRCs and advanced neoplasia cases not referred were expressed as percentages of participants with each target condition. False-positive referral reduction was the proportion of participants without the target condition who would not have been referred. All referral outcomes were modelled; no colonoscopies were withheld.

Exploratory descriptive subgroup analyses compared FIT118 alone with FIT100 plus RAID-CRC Screen by sex and age (50–59 and ≥60 years). Threshold and subgroup analyses were not prespecified and were interpreted as hypothesis-generating. Analyses were performed in Python (version 3.12.14; Python Software Foundation; https://www.python.org) using pandas and SciPy(version 1.17.0; SciPy Community; https://scipy.org/).

## 3. Results

### 3.1. Participant Flow and Baseline Characteristics

Among 1840 FIT-positive participants who underwent screening colonoscopy between December 2022 and October 2024, 162 did not meet the eligibility criteria, and 358 declined participation. Of 1320 enrolled participants, eight were excluded because of incomplete colonoscopy. Among the remaining 1312 participants, 21 had a non-evaluable RAID-CRC Screen result, leaving 1291 participants in the primary diagnostic accuracy analysis (Figure 1). All 1312 participants were retained in an additional intention-to-diagnose analysis of the referral strategy, in which non-evaluable results were assigned to colonoscopy referral. The final study closure report confirmed that informed consent was obtained for all analysed participants and reported no critical audit findings affecting scientific validity.

Median age was 62 years (IQR, 56–66), and 562 participants (43.5%) were women. Median FIT concentration was 226 ng Hb/mL (IQR, 145–527.5). CRC was detected in 34 participants (2.6%), advanced adenoma in 426 (33.0%), and advanced neoplasia in 441 (34.2%). Nineteen participants had both CRC and an advanced adenoma. RAID-CRC Screen was positive in 1087 participants (84.2%) and negative in 204 (15.8%) (Table 1).

### 3.2. Diagnostic Accuracy at the Programme FIT Threshold (Table 2)

RAID-CRC Screen identified 32 of 34 CRCs, corresponding to a sensitivity of 94.1% (95% CI, 80.3–99.3%). The test was negative in 202 of 1257 participants without CRC, yielding a specificity of 16.1% (95% CI, 14.1–18.2%), a PPV of 2.9% (95% CI, 2.0–4.1%), and an NPV of 99.0% (95% CI, 96.5–99.9%). The positive likelihood ratio was 1.121 (95% CI, 1.027–1.224), and the negative likelihood ratio was 0.366 (95% CI, 0.095–1.413).

For advanced neoplasia, 386 of 441 cases were RAID-CRC Screen-positive, and 55 were negative. Sensitivity was 87.5% (95% CI, 84.1–90.5%), specificity was 17.5% (95% CI, 15.0–20.3%), PPV was 35.5% (95% CI, 32.7–38.4%), and NPV was 73.0% (95% CI, 66.4–79.0%). The test correctly reclassified 149 of 850 participants without advanced neoplasia as negative, reducing false-positive FIT referrals for this endpoint by 17.5%.

An additional intention-to-diagnose analysis of the referral strategy included all 1312 participants with complete colonoscopy, assigning non-evaluable RAID-CRC Screen results to colonoscopy referral. There were 21 non-evaluable results (1.6%; 95% CI, 1.0–2.4%), including eight participants with advanced neoplasia and none with CRC. Under this strategy, 1108 participants would have been referred for colonoscopy, and 204 colonoscopies (15.5%; 95% CI, 13.6–17.6%) would have been avoided. Sensitivity and specificity were 94.1% and 15.8% for CRC, and 87.8% and 17.3% for advanced neoplasia, respectively (Table S1). The 204 participants who would not have been referred included two with CRC and 55 with advanced neoplasia, including those two CRCs.

**Table 2:** Diagnostic accuracy of RAID-CRC Screen among participants with a positive FIT at the programme threshold.

Target Condition	TP	TN	FP	FN	Sensitivity, % (95% CI)	Specificity, % (95% CI)	PPV, % (95% CI)	NPV, % (95% CI)	LLR + (95% CI)	LLR − (95% CI)
Colorectal cancer	32	202	1055	2	94.1 (80.3–99.3)	16.1 (14.1–18.2)	2.9 (2.0–4.1)	99.0 (96.5–99.9)	1.121 (1.027–1.224)	0.366 (0.095–1.413)
Advanced adenoma	372	150	715	54	87.3 (83.8–90.3)	17.3 (14.9–20.0)	34.2 (31.4–37.1)	73.5 (66.9–79.4)	1.056 (1.008–1.108)	0.731 (0.548–0.976)
Advanced neoplasia	386	149	701	55	87.5 (84.1–90.5)	17.5 (15.0–20.3)	35.5 (32.7–38.4)	73.0 (66.4–79.0)	1.061 (1.013–1.112)	0.711 (0.534–0.948)
Any colorectal neoplasia	696	83	391	121	85.2 (82.6–87.6)	17.5 (14.2–21.2)	64.0 (61.1–66.9)	40.7 (33.9–47.8)	1.033 (0.982–1.086)	0.846 (0.655–1.092)

CI, confidence interval; FN, false negative; FP, false positive; LLR, likelihood ratio; NPV, negative predictive value; PPV, positive predictive value; TN, true negative; TP, true positive.

### 3.3. Comparison with Alternative FIT Thresholds

Increasing the FIT threshold, alone or combined with RAID-CRC Screen, progressively reduced modelled colonoscopy referrals at the expense of lower CRC and advanced neoplasia detection (Table 3 and Table S2). FIT118 alone would have avoided 149 colonoscopies (11.5%), retaining all 34 observed CRCs but leaving 34 advanced neoplasia cases (7.7%) without referral. By comparison, FIT100 plus RAID-CRC Screen would have avoided 204 colonoscopies (15.8%), leaving two CRCs (5.9%) and 55 advanced neoplasia cases (12.5%) without referral.

At the highest threshold evaluated, FIT266 plus RAID-CRC Screen would have avoided 827 colonoscopies (64.1%), while 13 CRCs (38.2%) and 239 advanced neoplasia cases (54.2%) would not have been referred. Figure 2 illustrates the trade-off between colonoscopy reduction and CRC sensitivity across the evaluated strategies.

Labels indicate FIT thresholds in ng Hb/mL. Combined strategies required both FIT ≥ the specified threshold and a positive RAID-CRC Screen result. Analyses included 1291 participants with evaluable assay results and complete colonoscopy, including 34 with CRC. Colonoscopy reduction was calculated relative to referral of all participants under FIT100. Lines connect evaluated thresholds to aid visual interpretation. Corresponding 95% confidence intervals are reported in Supplementary Table S2.

### 3.4. Exploratory Subgroup Analyses

Exploratory analyses compared FIT118 alone with FIT100 plus RAID-CRC Screen according to sex and age (Supplementary Table S3). FIT118 retained all observed CRCs in each subgroup. FIT100 plus RAID-CRC Screen would have avoided more colonoscopies, but two CRCs would not have been referred, both in men aged under 60 years. Although all observed CRCs in women and participants aged ≥ 60 years were retained by both strategies, confidence intervals were wide. These descriptive findings do not establish safe subgroup-specific referral thresholds.

## 4. Discussion

In this external validation study embedded in the Galician organised CRC screening programme, RAID-CRC Screen showed sensitivities of 94.1% for CRC and 87.5% for advanced neoplasia among FIT-positive participants, with specificities of 16.1% and 17.5%, respectively. In the primary analysis, applying microbiota triage after FIT100 would have avoided 204 of 1291 colonoscopies (15.8%), while two CRCs and 55 advanced neoplasia cases, including those two CRCs, would not have been referred.

RAID-CRC Screen was designed to maximise sensitivity in an already FIT-positive population, and even a modest reduction in referrals could release endoscopy capacity. However, this potential benefit must be weighed against the clinical consequences of false-negative results: among the 204 participants who would not have been referred, 1.0% had CRC and 27.0% had advanced neoplasia. A negative result therefore did not reliably exclude clinically relevant lesions, and withholding colonoscopy could delay CRC diagnosis and leave advanced precursor lesions unresected. All participants underwent colonoscopy, so the safety and longer-term consequences of withholding the procedure were not evaluated. Assessment of clinical utility must consider these missed findings alongside implementation costs, turnaround time, non-evaluable results and the potential benefits of reallocating endoscopy capacity.

Our CRC sensitivity is consistent with the high values reported during development and prior screening evaluation. Malagón et al. reported CRC sensitivities of 94.7% in a 327-participant screening validation and approximately 95% in a subsequent clinical performance study [13,14]. In those studies, false-positive FIT reductions were approximately 16–20%, compared with 17.5% in Galicia. The difference may reflect variation in participant characteristics, lesion prevalence, sample processing, antibiotic or other environmental exposures, or the transportability of microbiome signatures across populations. Because the index test is qualitative and was applied without local recalibration, this study provides a relevant estimate of performance under the intended real-world pathway.

Comparison with FIT threshold adjustment provides a clinically relevant benchmark for assessing the added value of microbiota triage. The observed trade-offs indicate that reducing colonoscopy referrals must be considered alongside the loss of CRC and advanced neoplasia detection. These exploratory, descriptive comparisons do not establish superiority of either strategy, particularly given the limited number of CRC events and the selection and evaluation of alternative FIT thresholds within the same dataset. They therefore cannot identify an optimal or clinically safe referral threshold. Programme-level decisions require independent validation and assessment of local colonoscopy capacity, costs and longer-term clinical outcomes [8,9,10,11].

This study has several strengths. It represents a large external evaluation within an organised screening programme, using residual material from the routine FIT pathway, with colonoscopy and histopathology as the reference standard, and comparing the index test against clinically actionable FIT thresholds. Consecutive invitation and blinded interpretation reduce important sources of selection and review bias.

The limitations should be considered. First, only FIT-positive participants underwent colonoscopy, so the study estimates conditional performance within a selected population and cannot establish sensitivity or specificity for the entire invited population. Second, although the protocol-specified sample size was achieved, only 34 participants in the primary analysis had CRC, limiting the precision of the sensitivity estimate. The prespecified sensitivity target above 95% was not achieved, despite reaching the target reduction in modelled colonoscopy referrals. These findings do not establish the clinical safety of triage, and subgroup estimates should be interpreted cautiously. Third, the study evaluated diagnostic classification rather than actual prospective withholding of colonoscopy; the clinical outcomes of RAID-CRC Screen-negative participants remain modelled. Fourth, cost-effectiveness, participant acceptability, laboratory capacity, turnaround time, and management of invalid qPCR results were not evaluated. Finally, long-term follow-up is needed to quantify interval CRC risk among participants who would not be referred under a three-step strategy.

The results support further prospective assessment rather than immediate programme-wide implementation. A pragmatic impact study could randomise or phase the introduction of microbiota triage, monitor colonoscopy waiting times, evaluate safety-netting and repeat-testing pathways, and measure advanced neoplasia yield, interval cancers, costs, and participant-reported outcomes. Continuous calibration would be essential if quantitative FIT distributions or laboratory procedures change.

## 5. Conclusions

RAID-CRC Screen showed diagnostic performance broadly consistent with previous validation in FIT-positive screening participants. Microbiota triage and increasing the FIT threshold offered different trade-offs between colonoscopy reduction and lesion detection: modest threshold adjustment retained more lesions, whereas microbiota triage avoided more colonoscopies. These exploratory findings do not establish superiority or the clinical safety of either strategy. Their potential implementation requires prospective safety assessment and economic evaluation.

## Figures and Tables

**Figure 1:**
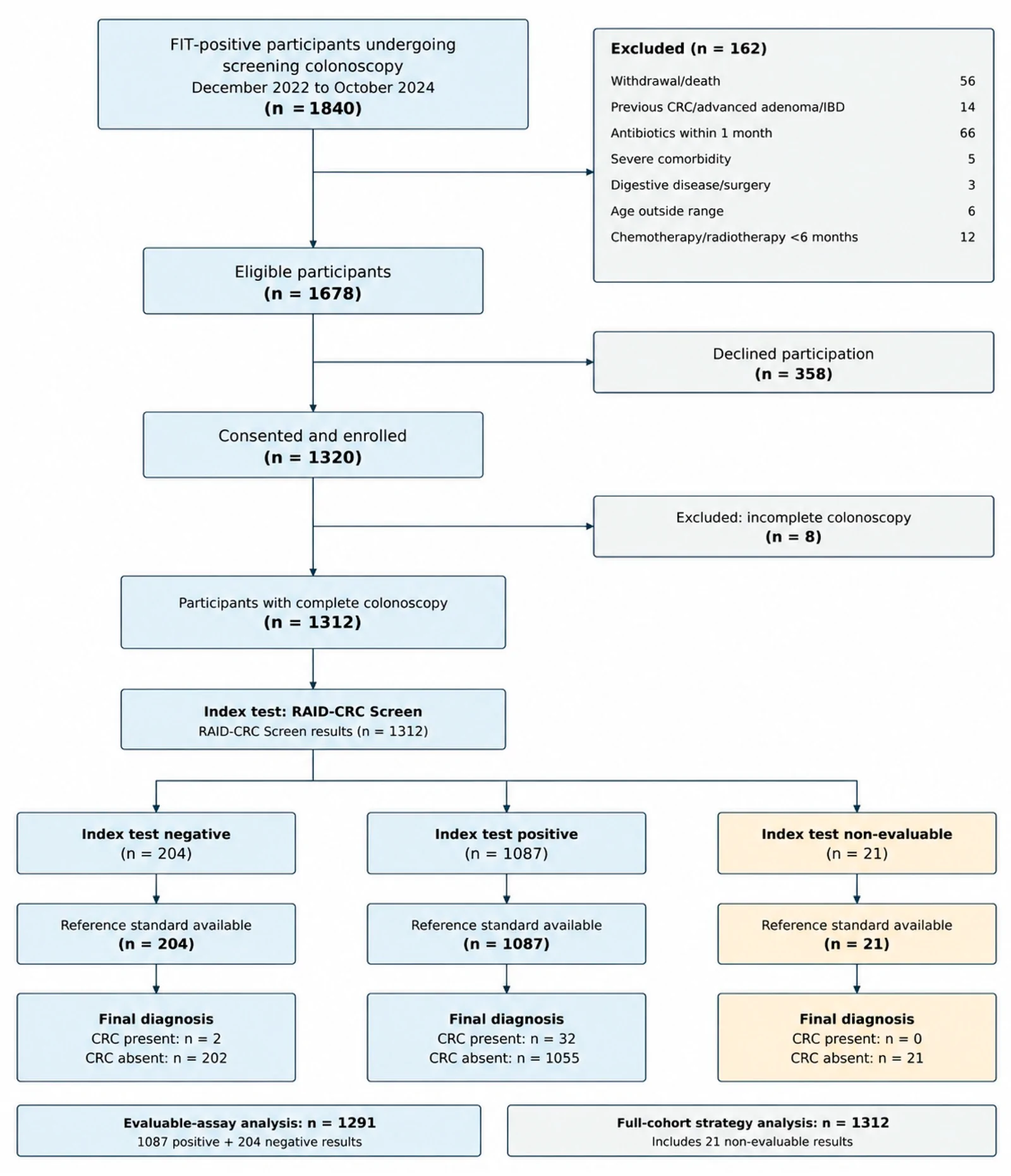
STARD-style participant flow diagram. CRC, colorectal cancer; FIT, faecal immunochemical test; IBD, inflammatory bowel disease.

**Figure 2:**
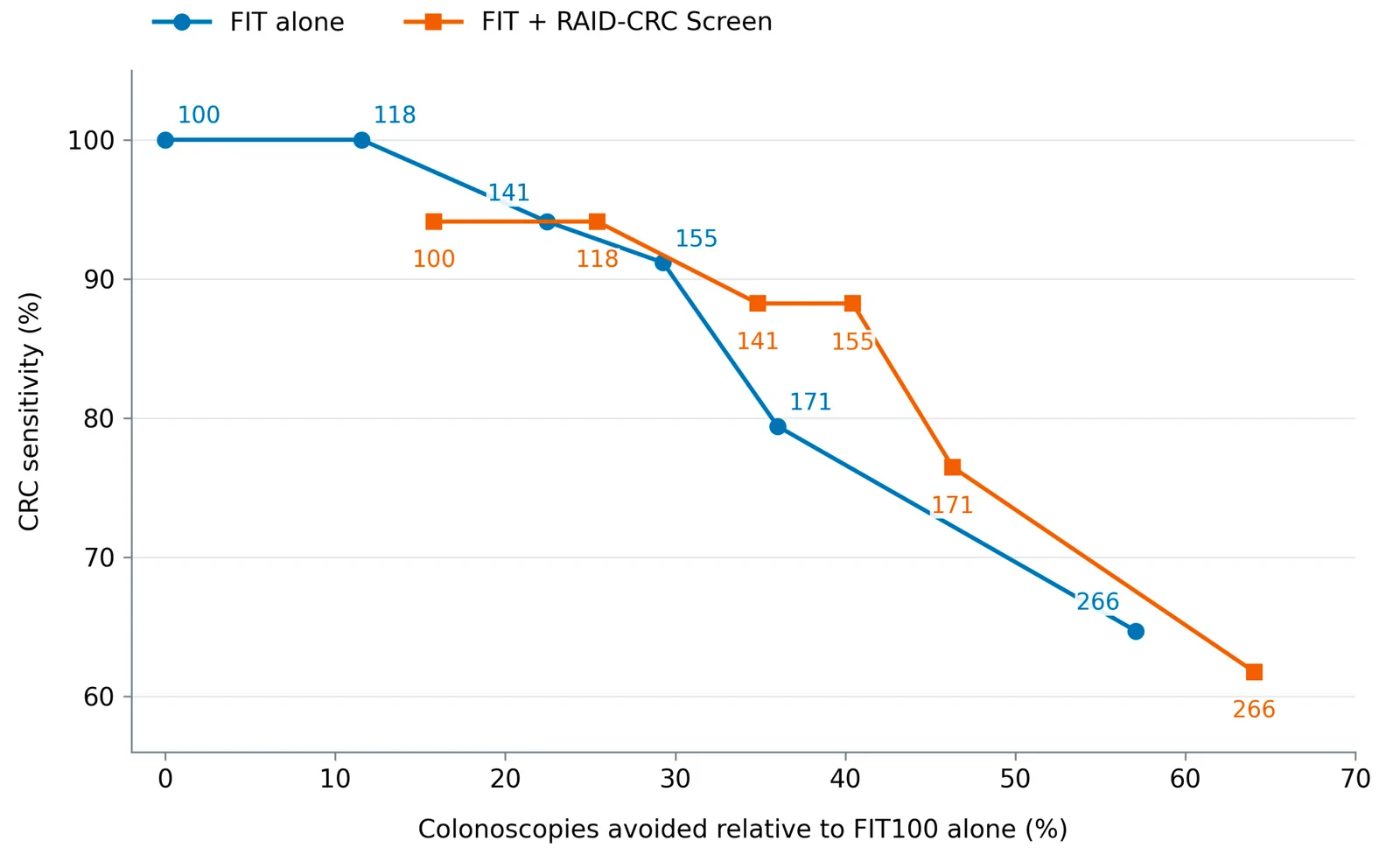
Trade-off between colonoscopy demand and CRC sensitivity for FIT threshold adjustment alone and FIT plus RAID-CRC Screen.

**Table 1:** Baseline demographic, screening, and colonoscopy characteristics of the analysed cohort.

Overall (n = 1291)	Characteristic
62 (56–66)	Age, median (IQR), years
562 (43.5)	Female sex, n (%)
226 (145–527.5)	FIT (ng Hb/mL) value, median (IQR)
34 (2.6)	Colorectal cancer, n (%)
426 (33.0)	Advanced adenoma, n (%)
441 (34.2)	Advanced neoplasia, n (%)
817 (63.3)	Colorectal neoplasia, n (%)
809 (62.7)	Adenomas, n (%)
1087 (84.2)	RAID-CRC Screen positive, n (%)

FIT, faecal immunochemical test; IQR, interquartile range.

**Table 3:** Estimated clinical impact of alternative FIT thresholds with and without RAID-CRC Screen triage.

FIT Cut-Off	Strategy	Colonoscopies Avoided, n (%)	CRCs Missed, n (%)	AN Missed, n (%)
100	FIT alone	0 (0.0)	0 (0.0)	0 (0.0)
100	FIT + RAID-CRC	204 (15.8)	2 (5.9)	55 (12.5)
118	FIT alone	149 (11.5)	0 (0.0)	34 (7.7)
118	FIT + RAID-CRC	328 (25.4)	2 (5.9)	87 (19.7)
141	FIT alone	290 (22.5)	2 (5.9)	65 (14.7)
141	FIT + RAID-CRC	450 (34.9)	4 (11.8)	115 (26.1)
155	FIT alone	378 (29.3)	3 (8.8)	91 (20.6)
155	FIT + RAID-CRC	522 (40.4)	4 (11.8)	137 (31.1)
171	FIT alone	465 (36.0)	7 (20.6)	114 (25.9)
171	FIT + RAID-CRC	598 (46.3)	8 (23.5)	159 (36.1)
266	FIT alone	737 (57.1)	12 (35.3)	212 (48.1)
266	FIT + RAID-CRC	827 (64.1)	13 (38.2)	239 (54.2)

## Data Availability

The participant-level data are not publicly available because they contain clinical information and are subject to European and Spanish data-protection requirements. De-identified data may be made available from the corresponding author upon reasonable request, subject to approval by the study sponsor and the relevant governance bodies.

## Supplementary Materials

The following supporting information can be downloaded at https://www.mdpi.com/article/10.3390/diagnostics16213655/s1. Supplementary Table S1: Intention-to-diagnose performance of the colonoscopy referral strategy among 1312 participants with complete colonoscopy. Supplementary Table S2: Diagnostic performance of alternative FIT thresholds with and without RAID-CRC Screen triage. Supplementary Table S3: Exploratory comparison of FIT118 alone and FIT100 plus RAID-CRC Screen by sex and age. STARD 2015 checklist, and the original study protocol in Spanish.
