# Supplementary material for: Diagnostic Accuracy and Colonoscopy-Sparing Potential of RAID-CRC Screen After a Positive Faecal Immunochemical Test
Source: Diagnostics (Basel). 2026 Nov 5;16(21):3655. doi: 10.3390/diagnostics16213655 (PMC13762127; doi:10.3390/diagnostics16213655)
Supplement: Supplementary file 1 [file diagnostics-16-03655-s001.zip › diagnostics-4614085-done-supplementary materials-main.pdf]

Supplementary Table S1. Intention-to-diagnose performance of the colonoscopy referral strategy among 1312 participants with complete colonoscopy.

Target condition	TP	TN	FP	FN	Sensitivity, % (95% CI)	Specificity, % (95% CI)	PPV, % (95% CI)	NPV, % (95% CI)
Colorectal cancer	32	202	1076	2	94.1 (80.3–99.3)	15.8 (13.8–17.9)	2.9 (2.0–4.1)	99.0 (96.5–99.9)
Advanced adenoma	380	150	728	54	87.6 (84.1–90.5)	17.1 (14.7–19.7)	34.3 (31.5–37.2)	73.5 (66.9–79.4)
Advanced neoplasia	394	149	714	55	87.8 (84.4–90.6)	17.3 (14.8–20.0)	35.6 (32.7–38.5)	73.0 (66.4–79.0)
Any colorectal neoplasia	710	83	398	121	85.4 (82.9–87.8)	17.3 (14.0–20.9)	64.1 (61.2–66.9)	40.7 (33.9–47.8)

Positive referral decisions comprised positive or non-evaluable RAID-CRC Screen results; negative referral decisions comprised negative results only. TP, TN, FP and FN refer to the referral strategy, not to the assay result alone. Confidence intervals were calculated using the exact Clopper–Pearson method.

Supplementary Table S2. Diagnostic performance of alternative FIT thresholds with and without RAID-CRC Screen triage.

FIT cut-off, ng Hb/mL	Strategy	CRC sensitivity, % (95% CI)	AN sensitivity, % (95% CI)	AN specificity, % (95% CI)	AN PPV, % (95% CI)	AN NPV, % (95% CI)
**100**	FIT alone	100.0	100.0	0.0	34.2 (31.6–36.8)	—
**100**	FIT + RAID-CRC	94.1 (80.3–99.3)	87.5 (84.1–90.5)	17.5 (15.0–20.3)	35.5 (32.7–38.4)	73.0 (66.4–79.0)
**118**	FIT alone	100.0 (89.7–100.0)	92.3 (89.4–94.6)	13.5 (11.3–16.0)	35.6 (32.9–38.5)	77.2 (69.6–83.7)
**118**	FIT + RAID-CRC	94.1 (80.3–99.3)	80.3 (76.2–83.9)	28.4 (25.3–31.5)	36.8 (33.7–39.9)	73.5 (68.3–78.2)
**141**	FIT alone	94.1 (80.3–99.3)	85.3 (81.6–88.4)	26.5 (23.5–29.6)	37.6 (34.6–40.6)	77.6 (72.3–82.3)
**141**	FIT + RAID-CRC	88.2 (72.5–96.7)	73.9 (69.6–78.0)	39.4 (36.1–42.8)	38.8 (35.5–42.2)	74.4 (70.2–78.4)
**155**	FIT alone	91.2 (76.3–98.1)	79.4 (75.3–83.0)	33.8 (30.6–37.1)	38.3 (35.2–41.6)	75.9 (71.3–80.2)
**155**	FIT + RAID-CRC	88.2 (72.5–96.7)	68.9 (64.4–73.2)	45.3 (41.9–48.7)	39.5 (36.1–43.1)	73.8 (69.8–77.5)
**171**	FIT alone	79.4 (62.1–91.3)	74.1 (69.8–78.2)	41.3 (38.0–44.7)	39.6 (36.2–43.0)	75.5 (71.3–79.3)
**171**	FIT + RAID-CRC	76.5 (58.8–89.3)	63.9 (59.3–68.4)	51.6 (48.2–55.1)	40.7 (37.0–44.5)	73.4 (69.7–76.9)
**266**	FIT alone	64.7 (46.5–80.3)	51.9 (47.2–56.7)	61.8 (58.4–65.0)	41.3 (37.2–45.6)	71.2 (67.8–74.5)
**266**	FIT + RAID-CRC	61.8 (43.6–77.8)	45.8 (41.1–50.6)	69.2 (66.0–72.3)	43.5 (39.0–48.2)	71.1 (67.9–74.2)

Analyses included 1291 participants with evaluable RAID-CRC Screen results and complete colonoscopy. Combined strategies required FIT ≥ the specified threshold and a positive RAID-CRC Screen result. Confidence intervals were calculated using the exact Clopper–Pearson method. All estimates are conditional on a positive programme FIT result (FIT ≥100 ng Hb/mL); FIT100 sensitivity and specificity reflect the cohort selection criteria rather than performance in the entire screening population. NPV was undefined for FIT100 alone because no participants tested negative. CRC, colorectal cancer; AN, advanced neoplasia; PPV, positive predictive value; NPV, negative predictive value.

Supplementary Table S3. Exploratory comparison of FIT118 alone and FIT100 plus RAID-CRC Screen by sex and age.

Subgroup	Participants, n	CRCs, n	Strategy	Colonoscopies avoided, n (%)	CRC sensitivity, % (95% CI)	AN sensitivity, % (95% CI)
Men	729	22	FIT118 alone	85 (11.7)	100.0 (84.6–100.0)	91.2 (87.3–94.1)
Men	729	22	FIT100 + RAID-CRC	105 (14.4)	90.9 (70.8–98.9)	87.8 (83.5–91.3)
Women	562	12	FIT118 alone	64 (11.4)	100.0 (73.5–100.0)	94.6 (89.6–97.6)
Women	562	12	FIT100 + RAID-CRC	99 (17.6)	100.0 (73.5–100.0)	87.1 (80.6–92.0)
50–59 years	489	14	FIT118 alone	53 (10.8)	100.0 (76.8–100.0)	92.8 (87.7–96.2)
50–59 years	489	14	FIT100 + RAID-CRC	71 (14.5)	85.7 (57.2–98.2)	88.6 (82.7–93.0)
≥60 years	802	20	FIT118 alone	96 (12.0)	100.0 (83.2–100.0)	92.0 (88.1–94.9)
≥60 years	802	20	FIT100 + RAID-CRC	133 (16.6)	100.0 (83.2–100.0)	86.9 (82.3–90.7)

Analyses included participants with evaluable RAID-CRC Screen results and complete colonoscopy. Combined triage required FIT ≥100 ng Hb/mL and a positive RAID-CRC Screen result. Colonoscopy reduction was calculated relative to referral of all participants within each subgroup. Advanced neoplasia was present in 294 men, 147 women, 166 participants aged 50–59 years and 275 aged ≥60 years. Confidence intervals were calculated using the exact Clopper–Pearson method. Sex and age classifications describe overlapping populations. CRC, colorectal cancer; AN, advanced neoplasia.
